# Dealkylation of Poly(methyl methacrylate) by TiCl_4_ Vapor Phase Infiltration (VPI) and the Resulting Chemical and Thermophysical Properties of the Hybrid Material

**DOI:** 10.1021/acs.chemmater.3c02446

**Published:** 2024-01-10

**Authors:** Shuaib A. Balogun, Sierra S. Yim, Typher Yom, Benjamin C. Jean, Mark D. Losego

**Affiliations:** School of Materials Science and Engineering, Georgia Institute of Technology, Atlanta, Georgia 30332,United States

## Abstract

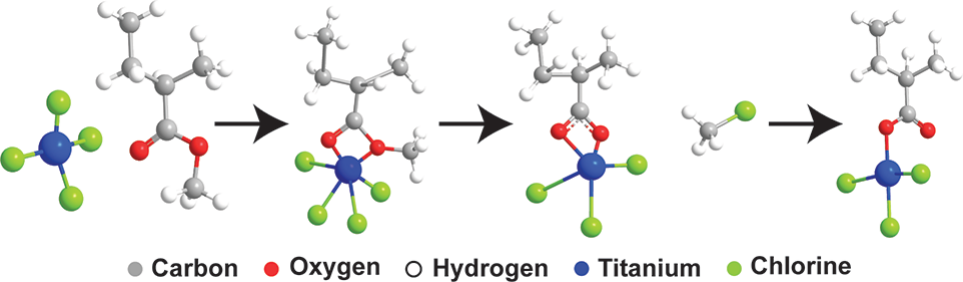

This study examines the chemical reaction pathways for vapor phase infiltration (VPI) of TiCl_4_ into poly(methyl methacrylate) (PMMA). VPI is a processing method that transforms organic polymers into organic–inorganic hybrid materials with new properties of interest for microelectronic patterning, technical textiles, and chemical separations. Understanding the fundamental chemical mechanisms of the VPI process is essential for establishing approaches to design the chemical structure and properties of these hybrid materials. While prior work has suggested that TiCl_4_ infiltration into PMMA does not disrupt the polymer’s carbonyl bond, a clear reaction mechanism has yet to be proposed. Here, we present a detailed X-ray photoelectron spectroscopy study that presents evidence for a concerted reaction mechanism that involves TiCl_4_ coordinating with the PMMA’s ester group to dealkylate the methyl side group, creating a chloromethane byproduct and primary chemical bonds between the organic and inorganic components of the hybrid material. Additional spectroscopy, quartz crystal microbalance gravimetry, and thermophysical and chemical property measurements of this material, including solubility studies and thermal expansion measurements, provide further evidence for this chemical reaction pathway and the subsequent creation of inorganic cross-links that network these TiO_*x*_–PMMA hybrid materials.

## Introduction

Vapor phase infiltration (VPI) infuses inorganic species into polymers by exposing the polymer to vapor-phase metal–organic or metal halide precursors that sorb into and become “entrapped” within the polymer.^1−7^ The resultant organic–inorganic hybrid materials have properties that differ from the pure polymer.^5,7−17^ For example, polymers and block copolymers infiltrated with TiO_*x*_ have been shown to have altered mechanical, optical, chemical, and thermal properties.^5,7,16−18^ The properties of these hybrids are dependent upon the quantity of infiltrated inorganic material, the chemical bonding between the organic and inorganic components, and the spatial distribution of the inorganic component.^2,8,19,20^

Infiltrated inorganic precursors become entrapped in polymers by chemically reacting with or adducting to the functional groups of the polymer. Adducted species may subsequently react with a sequentially delivered coreactant species to form a nonvolatile inorganic product that remains entrapped but possibly weakly bound to the polymer itself.^1,2,21^ For example, McGuinness et al.^2^ have shown that TMA infiltrated into PMMA can occur in both adducted and chemically reacted states, and this chemical binding state along with the inorganic’s spatial distribution directly impacts the resultant material properties. For example, at low temperatures, TMA sorbs into PMMA and forms a metastable reversible adduct, leading to weakly bound inorganic species that can actually be dissolved out of the hybrid by immersion in water, while at higher temperatures, the TMA is more likely to form primary chemical bonds to the carbonyl’s oxygen forming a strongly cross-linked network hybrid.^22^ The difference in this bonding chemistry leads to differences in the chemical stability of these hybrids in various solvents.^2^

In prior work, we have demonstrated that TiCl_4_ infiltration into PMMA occurs via a reaction-limited mechanism.^23^ Unlike TMA infiltration, TiCl_4_ infiltrates uniformly, albeit more slowly, within the entire depth of a PMMA thin film—that is to say, the TiCl_4_ rapidly diffuses into the PMMA, but entrapment is limited by the reaction rate. While this prior study clarified the rate-limiting step for the VPI process kinetics—the chemical reaction—the exact chemical mechanisms for this reaction process were not clarified. Multiple studies have hypothesized that the complexation of TiCl_4_ with the carbonyl of PMMA may be important.^24−26^ However, Biswas et al. have shown with *in situ* IR spectroscopy measurements that only weak interactions occur between TiCl_4_ and the carbonyl during both the TiCl_4_ and water dose.^24^ Thus, the exact chemical mechanisms for TiCl_4_ entrapment in PMMA during VPI are still unclear.

Although the entrapment mechanism is not fully understood, TiCl_4_ infiltrated polymers have been shown to have altered chemical, thermal, optical, and electrical properties.^5,7,12,16,17^ Additionally, PMMA/TiO_2_ nanocomposites formed from TiO_2_ nanoparticles have showed enhanced properties such as increased thermal stability, increased refractive index, and increased UV absorption.^27,28^ Thus, these TiO_*x*_/PMMA hybrids likely have potential interest for numerous applications.

In this study, systematic X-ray photoelectron spectroscopy (XPS) studies are used to track the behavior of PMMA’s ester group after varying times of TiCl_4_ infiltration. The changes in the oxygen spectrum provide particularly compelling evidence for a concerted chemical reaction that leads to dealkylation of the methoxy group and C–O–Ti bond formation. Additional chemical solubility data and thermal expansion measurements provide further evidence that a chemically cross-linked hybrid material is formed.

## Methods

### Materials

Poly(methyl methacrylate) (PMMA) with a molecular weight of approximately 75 kDa was acquired from Polysciences, Inc. A 5 wt % solution of the polymer in toluene (99.8% anhydrous, Sigma-Aldrich) was prepared. This solution was spun-cast onto silicon substrates at 3000 rpm for 30 s, resulting in films with a nominal thickness of 160–200 nm. Thicker films necessary for Fourier transform infrared (FTIR) spectroscopy were made using 15 wt % PMMA and cyclohexanone as the solvent. This solution was spun-cast onto double-side-polished silicon substrates at 6000 rpm for 60 s to achieve films of 1–1.2 μm. All films were then placed on a hot plate and heated to 150 °C for 1 h to remove any remaining solvent.

### Vapor Phase Infiltration

PMMA films were infiltrated in a custom-built reactor having a 28 L chamber and operated with decision-tree-based control software.^29^ PMMA was infiltrated at 90, 120, and 150 °C with TiCl_4_. The TiCl_4_ precursor was infiltrated with overpressures of ∼1 Torr. All pressures in the reaction chamber were measured with a Baratron capacitance manometer. All VPI processes used a single precursor–co-reactant cycle static hold scheme. The general process sequence was (1) ultrahigh purity N_2_ gas was flowed into the reactor to purge the system for 5 min, (2) the system was pumped down to base vacuum (30 mTorr) for an hour for full removal of water, (3) the chamber was isolated, (4) the TiCl_4_ precursor valve, which is connected directly to the chamber, was opened for 5 s to reach a vapor pressure of about 1 Torr of TiCl_4_, (5) the TiCl_4_ was then held in the chamber for between 1 and 48 h, (6) the system was then pumped to base vacuum for 5 min, (7) the water co-reactant valve, which is also connected directly to the chamber, was opened for 1 s to give a vapor pressure of 1.8 Torr in the chamber, and (8) the water was held in the chamber for 1 h before purging the system for 60 s and venting to the atmosphere.^23^

### *In Situ* Quartz Crystal Microbalance (QCM) Gravimetry Measurements

QCM experiments were performed in a hot-walled custom-built VPI reactor described elsewhere.^4,30^ The QCM used is a Phoenix high-temperature film thickness sensor PC-based system purchased from Colnatec. A polished gold 6 MHz RC quartz crystal was used as the substrate for the poly(methyl methacrylate). The crystal and the surrounding walls were heated to 150 °C under vacuum and flowing nitrogen to determine its baseline resonance frequency. Subsequently, 5 wt % solution of the PMMA in toluene was spun-cast onto the gold crystal substrate at 3000 rpm for 30 s, resulting in a film of ∼270 nm. The PMMA-coated crystal is then placed in the reactor and heated to 150 °C under vacuum and flowing nitrogen to determine the resonance frequency with just the bare polymer. VPI is then conducted as described above except for three key differences: (1) the TiCl_4_ precursor valve, which is connected directly to the chamber, was opened for 0.5 s to reach a vapor pressure of about 3.2 Torr of TiCl_4_ due to the smaller size of this reactor, (2) the film is pumped to base vacuum for 24 h between the TiCl_4_ dose and the water dose to ensure all unreacted precursor and byproducts have sufficient time to escape the polymer, and (3) the water co-reactant valve, which is also connected directly to the chamber, was opened for 0.5 s to give a vapor pressure of 12 Torr in the chamber. The crystal frequency was recorded every second during this time and was exported and converted to mass via the Sauerbrey equation.^31^

### Residual Gas Analyzer (RGA)

An EXtorr XT Series residual gas analyzer operated on a PF70 turbomolecular vacuum pump was attached to the hot-walled, custom-built VPI reactor described above via a capillary tube to sample the reaction atmosphere. The mass/charge (*m*/*z*) was determined with a scan speed of 48/s from 1 to 200 atomic mass units. The gas atmosphere was sampled by (1) opening a valve that transmits the gas in the reactor to the RGA and (2) turning on the filament inside the RGA and analyzing the gas atmosphere.

### Fourier Transform Infrared Spectroscopy

Attenuated total reflectance Fourier transform infrared (ATR-FTIR) spectroscopy was performed on 1 μm films of PMMA on double-sided polished, low-conductivity silicon. The spectra were collected on a Thermo Scientific Nicolet iS5 FTIR spectrometer with an iD7 ATR accessory and a diamond crystal. Spectra were collected with a resolution of 4 cm^–1^ and are the average of 64 scans.

### X-ray Photoelectron Spectroscopy (XPS)

XPS was performed by using a Thermo K-alpha system using a monochromatic Al Kα X-ray source (1486.6 eV) with a 60° incident angle and a 90° emission collection geometry. Survey scans were conducted at a pass energy of 200 eV and for binding energies from −10 to +1350 eV. For the elemental analysis, the following elements at the following binding energies were collected: Ti 2p (448–475 eV), O 1s (525–545 eV), C 1s (279–298 eV), Cl 2p (190–210 eV), and Si 2p (95–110 eV). Films were etched with a raster size of 400 × 400 μm^2^, an ion gun voltage of 2000 V, a high current, and for 65 s, yielding an approximate rate of 25 nm per etch level. At each level the elemental analysis and survey scan was performed. Three survey scans were conducted for all elements at each etch level. A Shirley background subtraction was used to determine the atomic percentage of Ti while a simple background subtraction was used for determining other atomic percentages.

### Spectroscopic Ellipsometry and Measurement of CTE and Glass Transition Temperatures

Film thicknesses were measured with an Alpha-SE spectroscopic ellipsometer (J.A. Woollam) at a 70° angle over a spectral range of 340–900 nm. The film was measured before and after infiltration to determine the starting and ending film thicknesses and refractive index (at a wavelength of 632.8 nm). A Cauchy model was used to ascertain the film thickness and refractive indices. The *A* and *B* Cauchy coefficients and thicknesses were fitted. This layer was stacked on a 2.3 nm layer of native SiO_2_ on a silicon substrate.

The coefficient of thermal expansion (CTE) and the glass transition temperature (*T*_g_) were determined by measuring the polymer thickness over a range of temperatures (40–150 °C). Details of this measurement setup can be found in prior studies.^8,32,33^ Measurements were taken at 5 °C intervals while the film was kept at a constant temperature for at least 10 s. No discernible differences in film thickness occurred for repeated measurements beyond 10 s at a given temperature. Films remained stationary throughout each temperature excursion, such that the exact spot was continuously measured. The volumetric CTE was determined from the slope of the thickness versus temperature. Assuming constrained isotropic behavior, the volumetric CTE value was divided by 3 to determine the more commonly reported linear CTE.

### Dissolution in Toluene

Films were placed in 10 mL vials of toluene, which is a good solvent for the pure PMMA polymer. Films were removed from solution at various time intervals, placed in a fume hood for 5 min to dry, and then had their thickness measured with spectroscopic ellipsometry to determine the extent of film dissolution.

## Results and Discussion

### Chemical Mechanism

**Figure 1:**
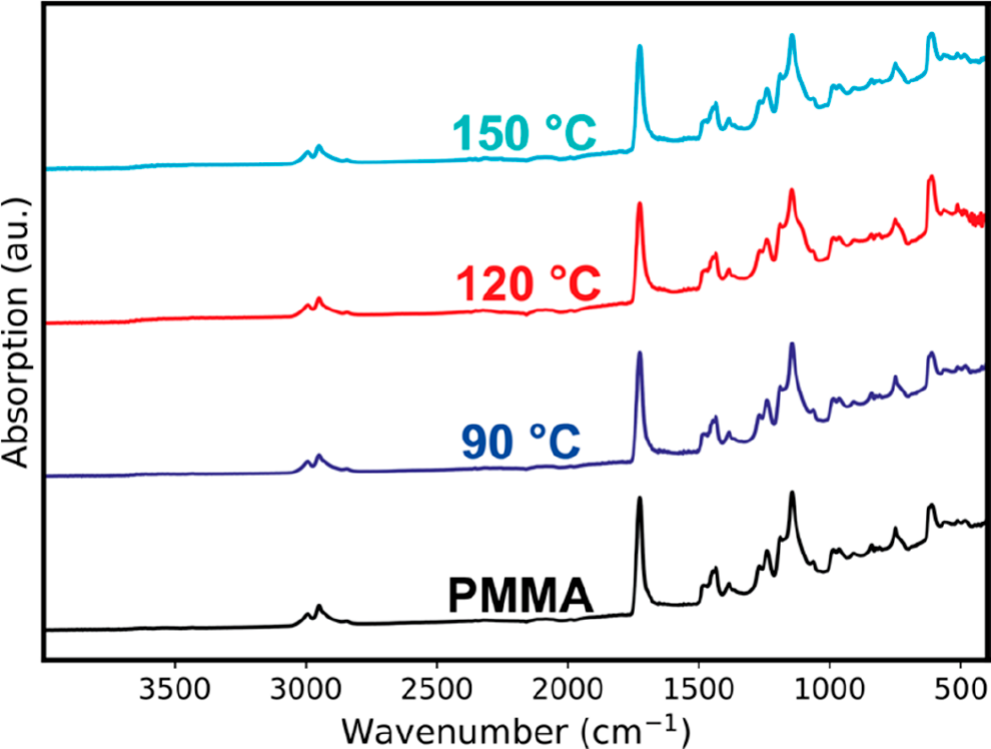
FTIR spectra for neat PMMA and PMMA infiltrated with TiCl_4_ + H_2_O at varying temperatures (90, 120, and 150 °C) with 24 h precursor exposure time.

Previously, we have used the spatiotemporal profiles of the infiltrated TiO_*x*_ inorganics to determine that the TiCl_4_–PMMA VPI process proceeds via a reaction-limited mechanism.^23^ Here, we attempt to identify the chemical reaction that constitutes this rate-limiting step. Figure 1 presents data collected from IR studies completed before and after VPI using a 24 h TiCl_4_ exposure step at 90, 120, and 150 °C. The FTIR spectrum for neat PMMA shows the expected C=O stretch at 1729 cm^–1^, a C–C–O stretch at 1240 cm^–1^, a C–O–R stretch at 1145 cm^–1^, a symmetric C–H stretch of CH_3_ at 2954 cm^–1^, a C–H stretch of CH_2_ at 2994 cm^–1^, and a C–H bending of CH_3_ and CH_2_ at 1440 and 1480 cm^–1^.^34−36^ Above this spectrum are the spectra for the infiltrated hybrid at varying process temperatures. Difference spectra are also reported in Figure S1 of the Supporting Information. In all cases, only minor changes are observed in the infiltrated spectra. Specifically, the carbonyl stretch shows little to no bleaching upon infiltration, suggesting little to no direct reaction of this functional group with TiCl_4_ and an end product that contains carbonyls. This observation of undisturbed carbonyl groups when infiltrating with TiCl_4_ is consistent with the *in situ* IR data previously reported by Biswas.^24^ Additionally, the absorption for the C–O–R stretch is also largely unchanged. This result is significantly different from what is understood for trimethylaluminum (TMA) infiltration into PMMA, where the carbonyl stretch is completely quenched and the C–O–R stretch shows a significant frequency shift at infiltration temperatures above ∼100 °C, indicative of a chemical reaction between the TMA and ester group.^1,2,24,37,38^ While the small amount of C=O quenching and C–O–R vibrational shifts detected here in the difference spectra could indicate some amount of direct reaction between TiCl_4_ and PMMA’s carbonyl group at the higher process temperatures, these results largely suggest that some other chemical reaction mechanism is dominant for the TiCl_4_/PMMA VPI chemistry.

**Figure 2:**
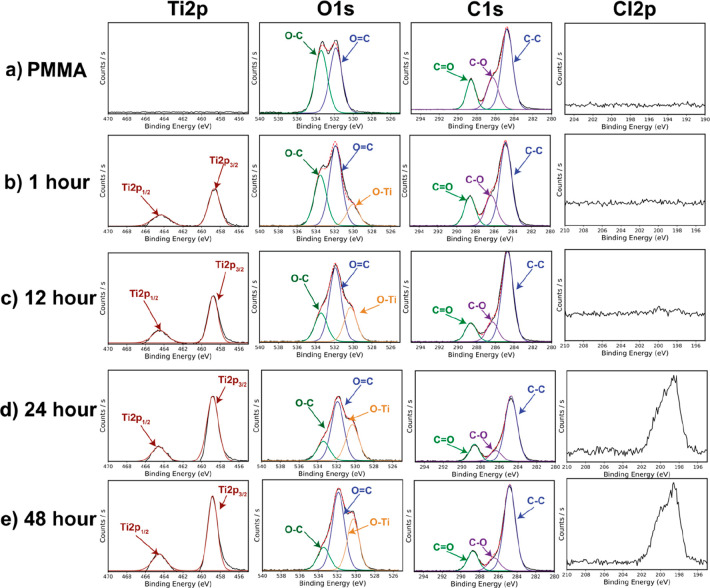
XPS spectra for PMMA films infiltrated with TiCl_4_ + H_2_O at varying TiCl_4_ precursor exposure times (hours) of (a) 0 (neat PMMA), (b) 1, (c) 12, (d) 24, and (e) 48 h. All VPI was done at 150 °C. The Ti 2p, O 1s, C 1s, and Cl 2p spectra are shown for each infiltration time. Emission intensity axes (abscissas) are kept constant down each column (i.e., for each element). Deconvolutions are labeled for the Ti, O, and C spectra.

Figure 2a–e presents XPS spectra for PMMA films infiltrated with TiCl_4_ for 0 (neat PMMA), 1, 12, 24, and 48 h at 150 °C. Note that these are collected from the film’s surface. Surface scans were used because the surface is assumed to be the most saturated portion of the infiltrated polymer.^1,4,21^ As expected, no Ti is detected in the uninfiltrated PMMA film with zero counts observed (Figure 2a). Upon infiltration (Figure 2b–e), a clear doublet emerges at 458.7 and 464.45 eV that is consistent with Ti in its 4+ oxidation state. The intensity of these Ti emissions also generally increases with TiCl_4_ exposure time, consistent with the expected increase in the titanium loading. The second column of Figure 2 shows the evaluation of the O 1s spectrum with TiCl_4_ exposure time; these data are particularly indicative of the chemical mechanism. For neat PMMA, near equal amounts of the methyl–oxy (C–O) at 533.6 eV and carbonyl (C=O) at 530.5 eV are observed as expected. However, with infiltration, the methyl–oxy bond decreases in intensity while the carbonyl bond maintains its intensity. This C–O intensity continues to reduce as exposure time continues to increase. Concurrently, a metal–oxide (Ti–O) emission emerges at 530.5 eV and increases with TiCl_4_ exposure time, consistent with the formation of infiltrated inorganics. These changes in the O 1s spectra suggest that with increasing TiCl_4_ exposure times, the C–O species are being consumed, while Ti–O bonds are being created. A similar relative reduction of the C–O species is observed in the C 1s spectra at 286.3 eV, particularly evident at exposure times of 24 and 48 h. At these long hold times, the ratio of C–O to C=O (288.5 eV) significantly reduces, again suggesting a consumption of C–O bonds. Lastly, the Cl 2p emission is also reported. Negligible Cl is detected until 24 h of TiCl_4_ exposure, at which point the signal is still relatively weak (0.12–0.60 at. %) and difficult to deconvolute. We can roughly attribute the Cl to possibly a Ti–Cl and/or a C–Cl state, which have been reported to occur at 198.5–199 and 200 eV, respectively.^40^ These may suggest a subsequent reaction that occurs after a long time or an inability to fully hydrolyze some of TiCl_4_ when inorganic loading becomes high.

**Figure 3:**
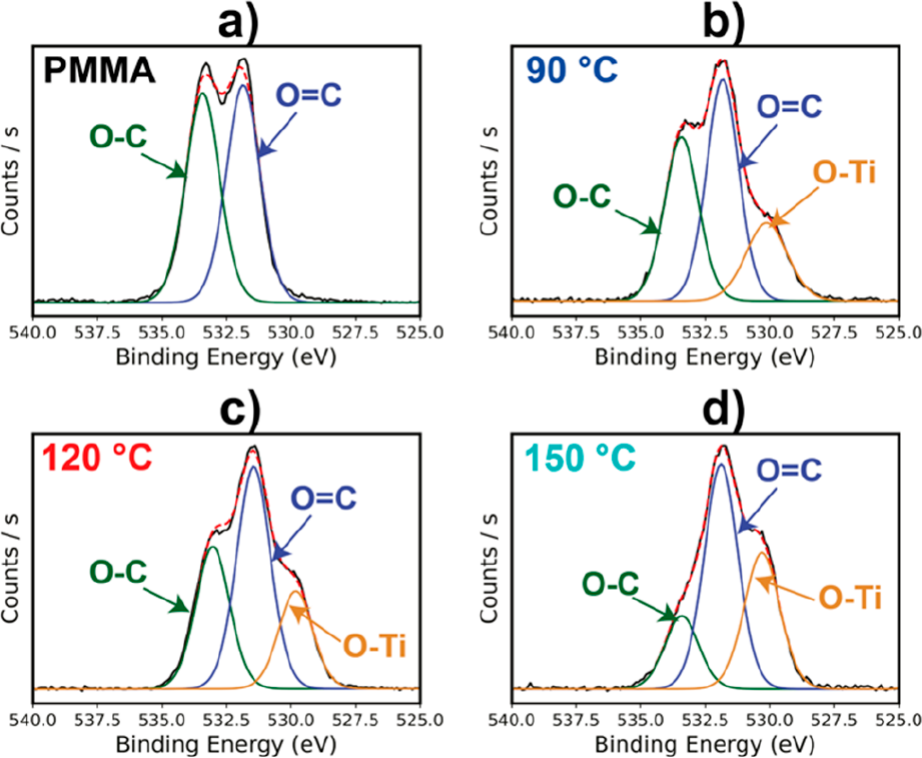
O 1s XPS plots for PMMA films infiltrated with TiCl_4_ + H_2_O at varying processing temperatures: neat PMMA, 90 °C, 120 °C, and 150 °C. Processes were performed with 24 h TiCl_4_ exposure time.

To further confirm these chemical trends, XPS spectra were also collected for hybrids created at varying VPI process temperatures and a fixed TiCl_4_ exposure time of 24 h, comparable to the FTIR data reported in Figure 1. Presumably, the temperature would increase the reaction kinetics for such a reaction. Figure 3 plots the O 1s XPS spectra for this series of films. For neat PMMA (Figure 3a), equal amounts of the methyloxy (C–O) and carbonyl (C=O) are observed, as expected. At the lowest process temperature (90 °C, Figure 3b), the C–O emission decreases, while Ti–O emission increases. This trend then continues with increasing temperature (120 and 150 °C), similar to what is observed as a function of exposure time in Figure 2.

**Scheme 1:**
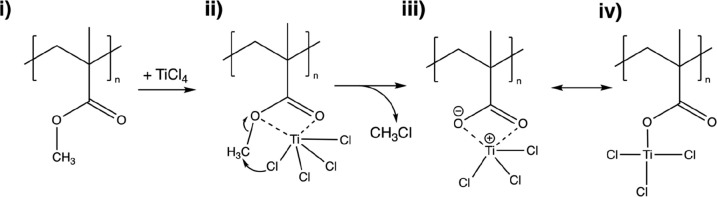
Proposed Reaction Mechanism for TiCl_4_ Infiltration of PMMA The reaction mechanism displays a Lewis acid concerted dealkylation reaction between the TiCl_4_ and carbonyl of the PMMA, resulting in a chloromethane byproduct and a substituted methyl group.

Overall, these spectroscopic observations suggest a chemical reaction that disrupts the methoxide side group of PMMA but leaves the carbonyl functionality intact. Both the reduction of the intensity of the O–C peak in the O 1s spectra and the reduction of the intensity of the C–O peak in the C 1s spectra suggest the loss of the methyl group. Metal chlorides have been shown to readily form adducts with ester groups via bidentate binding between the metal and the ester. These adducts can then drive concerted reactions.^41^Scheme 1 proposes a similar interaction between PMMA and TiCl_4_ that is consistent with these spectroscopic observations. The proposed mechanism follows a concerted dealkylation pathway between the TiCl_4_ and the ester moiety of PMMA, resulting in a chloromethane byproduct. Because TiCl_4_ is a known Lewis acid, TiCl_4_ can conceivably adduct with the lone pair electrons from the ester group, creating a TiCl_4_:PMMA complex (ii). We speculate that this complex may place the Ti in an octahedral coordination, which is common for Ti, bonding to its four chlorine ligands and the two oxygens of the ester group. In this configuration one of the chlorines in the basal plane of the oxygens would be aligned with the methyl group, making it susceptible to reaction. Subsequently, this Cl^–^ ion attacks the Me–O bond of the ester group, dealkylating it as a chloromethane byproduct and leaving a negatively charged carboxylate group adducted to a positively charged TiCl_3_^+^ (iii). These two charged species presumably form a more permanent primary bond (iv) between Ti and one of the oxygens, although a resonance between (iii) and (iv) and a switching between which O the Ti is bonded to cannot be precluded. Interestingly, this proposed mechanism results in a residual carbonyl group, consistent with IR and XP spectroscopic observations, and the small to negligible shift in the C–O–R vibration in IR spectroscopy is consistent with the similar vibrational frequencies reported for both C–O–R and C–O–M species.^39^ In general, the alkyl cleavage of esters to form carboxylic acids is well documented in the literature.^42−45^ This process commonly occurs by acid- or base-catalyzed hydrolysis of esters following a concerted dealkylation mechanism. Reports exist for alkyl cleavage of esters via strong Lewis acids such as AlCl_3_, TiCl_4_, or other metal halides as a catalyst in the presence of another co-reactant.^41,46^ However, to our knowledge, no reports exist for dealkylation of esters in polymers using gaseous metal halides in low vapor pressure environments. Here we posit that it is this dealkylation reaction that acts as the rate-limiting step for the TiCl_4_–PMMA VPI process.

**Figure 4:**
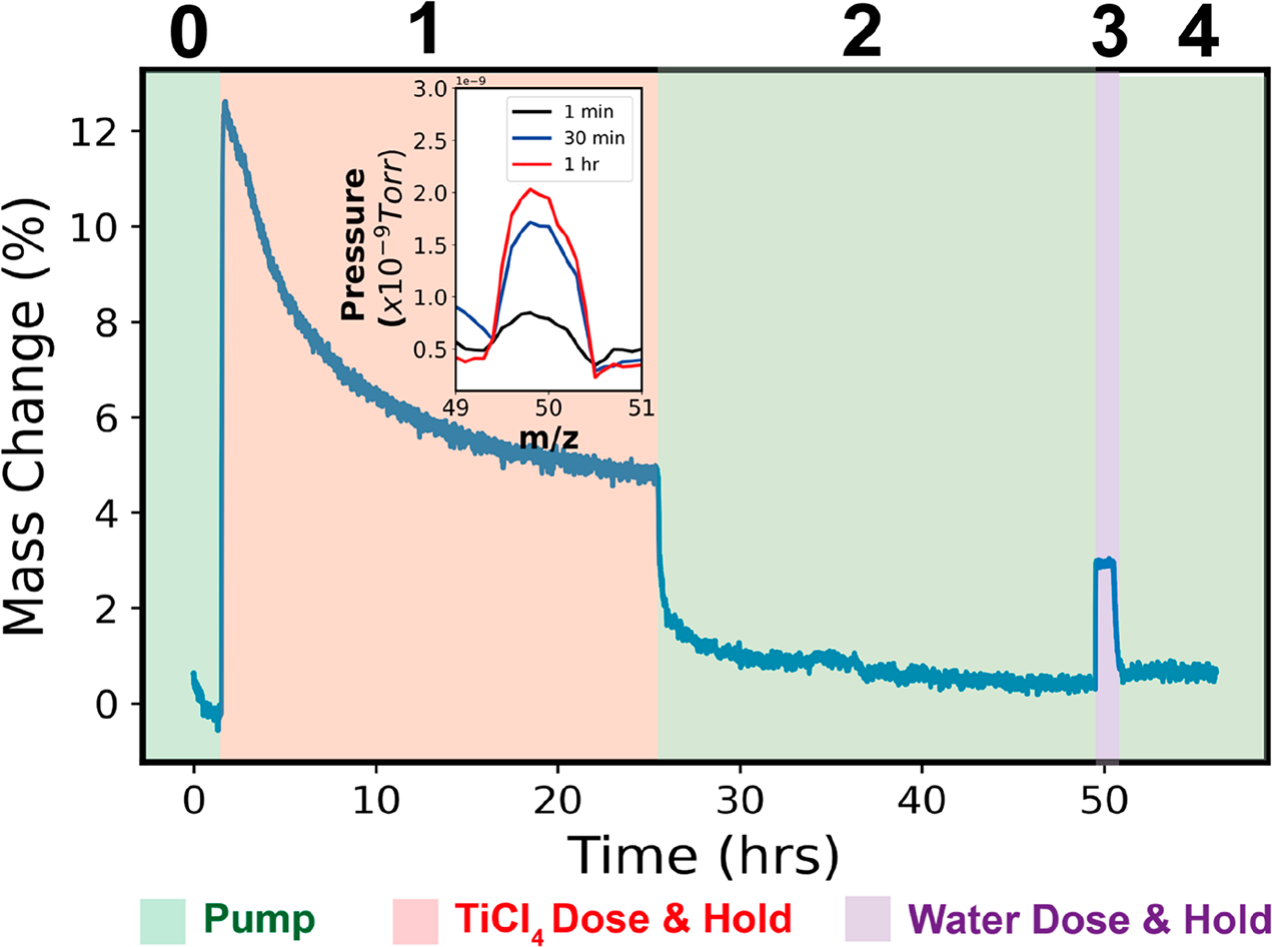
Mass uptake versus time plot collected via *in situ* QCM gravimetry during VPI of TiCl_4_ + H_2_O into PMMA at 150 °C. The plot is separated into five temporal regimes: (0) preinfiltration pumping (vacuum base pressure), (1) TiCl_4_ exposure (3.2 Torr), (2) TiCl_4_ removal via vacuum pumping, (3) H_2_O exposure (12 Torr), and (4) H_2_O removal via vacuum pumping. The mass uptake is normalized to the original mass of the polymer (3.24 × 10^–05^ g) to provide a percentage of mass added to the polymer via infiltration. All masses are calculated from the Sauerbrey equation.^31^ The inset shows *in situ* RGA data collected during step 1 that provides the gaseous composition of the atmosphere of the VPI chamber after 1 min, 30 min, and 1 h of TiCl_4_ exposure.

To further validate this proposed mechanism, Figure 4 presents quartz crystal microbalance (QCM) gravimetry analysis of this TiCl_4_ + H_2_O – PMMA VPI process collected at 150 °C. While an in-depth analysis of this data is beyond the scope of this paper, a brief quantitative and qualitative assessment provides further evidence supporting the chemical pathway proposed in Scheme 1. The QCM data are separated into five regions: (0) preinfiltration pumping, (1) TiCl_4_ exposure (24 h), (2) TiCl_4_ removal via vacuum pumping (24 h), (3) H_2_O exposure (1 h), and (4) H_2_O removal via vacuum pumping. In the initial stage of TiCl_4_ exposure, a rapid increase in mass of approximately 4.00 × 10^–6^ g is observed consistent with sorption of TiCl_4_ into PMMA. Interestingly, though, within 5 min, a maximum mass uptake is reached, and the mass begins to decrease. This decrease in mass suggests the loss of a high-mass byproduct, such as chloromethane, consistent with the chemical pathway proposed in Scheme 1. After about 20 h of TiCl_4_ exposure, mass loss nearly stops, and a constant mass is reached, suggesting the reaction has ended. The total mass loss during the hold step is approximately 2.40 × 10^–6^ g, which we attribute to chloromethane byproduct loss. Subsequently, upon TiCl_4_ removal via vacuum pumping, the mass decreases again quite rapidly, presumably due to the desorption of any unreacted TiCl_4_ that is dissolved only in the polymer and not chemically bound to the polymer. The total mass of desorbed species is 1.28 × 10^–6^ g. Removal of additional byproducts may also happen in this step. However, we assume that only unreacted TiCl_4_ desorbs. This removal of species occurs quickly, with most mass loss occurring within less than 1 h of pumping. Then upon H_2_O exposure, a mass rise is observed, presumably due to sorption of water. However, interestingly, upon water removal via vacuum pumping, the mass returns to nearly the same value as that prior to the water exposure; only a small amount of mass is gained, ∼0.25%. While this result may occur because a reaction species has the same mass as a byproduct species, this explanation seems unlikely given that chlorine-containing byproducts have considerably higher atomic masses than hydroxyl or oxide linkages. Thus, the more reasonable explanation appears to be that the water is just fully desorbing from the hybrid film without reacting with anything inside the film. This suggests that the TiCl_4_ is “fully reacted” with the polymer prior to the water exposure step, and no further hydrolysis occurs upon water exposure. The lack of observable hydroxide groups in the FTIR spectra of Figure 1 provides further evidence that few hydrolysis reactions are occurring in this step.^47^

Using the masses that we described above, the mass of TiCl_4_ that is consumed during the hold step and remains entrapped in the hybrid is 2.72 × 10^–6^ g, which equates to reacting 1.43 × 10^–8^ mol of TiCl_4_. Assuming that the mass loss is chloromethane with a molar mass of 50.5 g/mol, this equates to a molar loss of 4.75 × 10^–8^ mol of CH_3_Cl. The molar ratio of TiCl_4_ reacted to the CH_3_Cl released is 1:3.32. This result indicates that each of the entrapped TiCl_4_ molecules reacts with on average ∼3.3 methoxy functional groups or forms ∼3.3 bonds to PMMA chains. This value approaches the full reaction extent of 4 bonds, and we suspect steric hindrances limit any further reaction under the process conditions explored. For a more detailed explanation of these calculations, refer to Figure S3.

Additionally, in preliminary efforts to sample the gas atmosphere during TiCl_4_ vapor phase infiltration with a residual gas analyzer (RGA), we have been able to detect a signal at a mass-to-charge ratio of 50 units, which is consistent with the fractionation behavior expected for a chloromethane byproduct. This signal, shown in the inset of Figure 4, is observed to increase with the infiltration time, providing further evidence for Scheme 1. We do note that we cannot preclude the involvement of the HCl byproducts in this reaction mechanism, and that it is possible that HCl may serve as a catalyst for the proposed mechanism. However, at least in preliminary testing we did not find any direct reaction of PMMA with aqueous HCl solutions at elevated temperatures nor did attempts to reduce the HCl byproduct in the chamber during VPI alter the amount nor rate at which inorganic infiltration occurred.

### Chemical and Thermophysical Properties of TiO_*x*_–PMMA Hybrids

**Figure 5:**
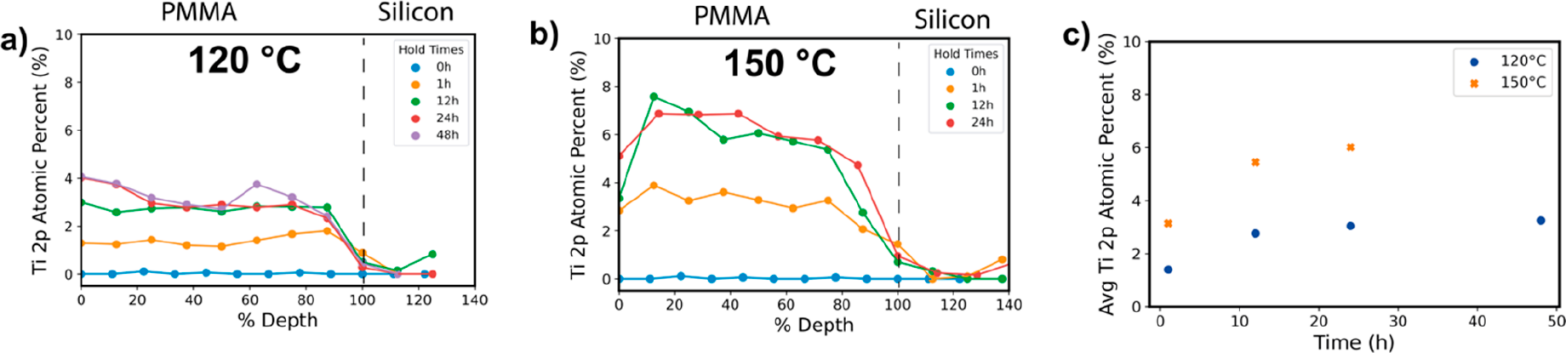
XPS depth profiles collected from PMMA films infiltrated with TiCl_4_ + H_2_O at varying precursor exposure times from 0 h (neat PMMA) to 48 h. (a) Processes were performed at 120 °C. (b) Processes were performed at 150 °C. (c) Average Ti 2p atomic percentage for infiltration of TiCl_4_ + H_2_O occurring at infiltration temperatures of 120 and 150 °C. The average Ti 2p atomic percentage was calculated using the weighted average of XPS atomic percentage values from (a) and (b). All films are nominally 200 nm thick, but the depth is normalized to the silicon substrate signal (not shown for clarity).

We have subsequently interrogated the chemical and thermophysical properties of the TiO_*x*_–PMMA hybrid materials because these properties often give physical insights into the hybrid’s chemical structure. These properties often provide evidence of whether chemical cross-links, via primary chemical bonds, are formed between the organic and inorganic constituents. Specifically, we have investigated the TiO_*x*_–PMMA hybrids prepared at infiltration temperatures of 120 and 150 °C at varying hold times because these films are expected to be fully infiltrated throughout the entire film depth.^23^Figure 5 presents XPS depth profiling data for 120 °C (Figure 5a) and 150 °C (Figure 5b) infiltration into 200 nm PMMA films at varying exposure times. The abscissa axis has been normalized to the total film thickness based upon the silicon substrate signal to improve comparisons among each film. At 120 °C (Figure 5a) Ti is uniformly present throughout the entire depth, and the concentration increases from ∼1.5 to ∼3 at. % as the TiCl_4_ exposure time is increased from 1 to 12 h. Above 12 h of exposure time, the inorganic loading approximately saturates.^23^Figure 5a corroborates the reaction-limited profile observed in our previous paper at 135 °C; however, we observe a lower overall titanium concentration due to the lower processing temperature.^23^ A similar trend is observed at a VPI process temperature of 150 °C (Figure 5b). However, notably, only 1 h of TiCl_4_ exposure is needed at 150 °C to reach the same inorganic loading as is achieved after 12 h of exposure at 120 °C (∼3.5%). At 12 and 24 h of exposure time, inorganic loading saturates at ∼6.5 at. % Ti. To establish structure–property relationships, we calculated average Ti 2p atomic percentages from the depth profiles of Figure 5. Figure 5c plots these average percentages for TiCl_4_ infiltration at 120 and 150 °C as a function of exposure times.

**Figure 6:**
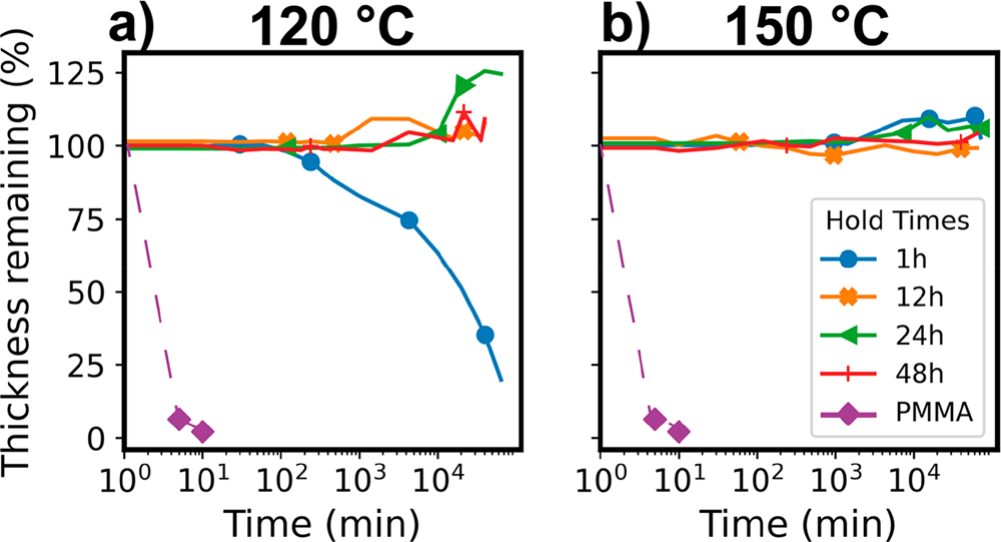
Dissolution plots for PMMA films infiltrated with TiCl_4_ + H_2_O at varying precursor exposure times from 0 h (neat PMMA) to 48 h. Tracking the % thickness remaining at different time points over 4 weeks. (a) Processes were performed at 120 °C. (b) Processes were performed at 150 °C. The *x*-axis is presented in log scale.

Figure 6 plots the dissolution behavior of these hybrid materials as a function of the immersion time in toluene, a good solvent for the pure PMMA polymer. The total immersion time here is 4 weeks. As expected, the untreated PMMA films fully dissolve within 10 min. In Figure 6a, we observe that films infiltrated at 120 °C have enhanced chemical stability at all exposure times. Hybrids synthesized from 1 h of VPI exposure, which is ∼1.5 at. % Ti, take >100 min before dissolution begins, but these films do approach full dissolution after ∼4 weeks of immersion. Hybrids prepared at 120 °C and 12 h of VPI exposure show no evidence for dissolution for up to 4 weeks of immersion in toluene, indicating good chemical stability. These hybrids, especially those prepared at shorter infiltration times (12 and 24 h), do show some swelling at long immersion times, which is indicative of a cross-linked organogel. Higher degrees of cross-linking would be expected to reduce the extent of swelling observed.^8^Figure 6b plots the dissolution behavior in toluene for TiO_*x*_–PMMA hybrids prepared at 150 °C. Here, all hybrids remain stable through 4 weeks of immersion, with minimal swelling, suggesting even more substantial cross-linking.

**Figure 7:**
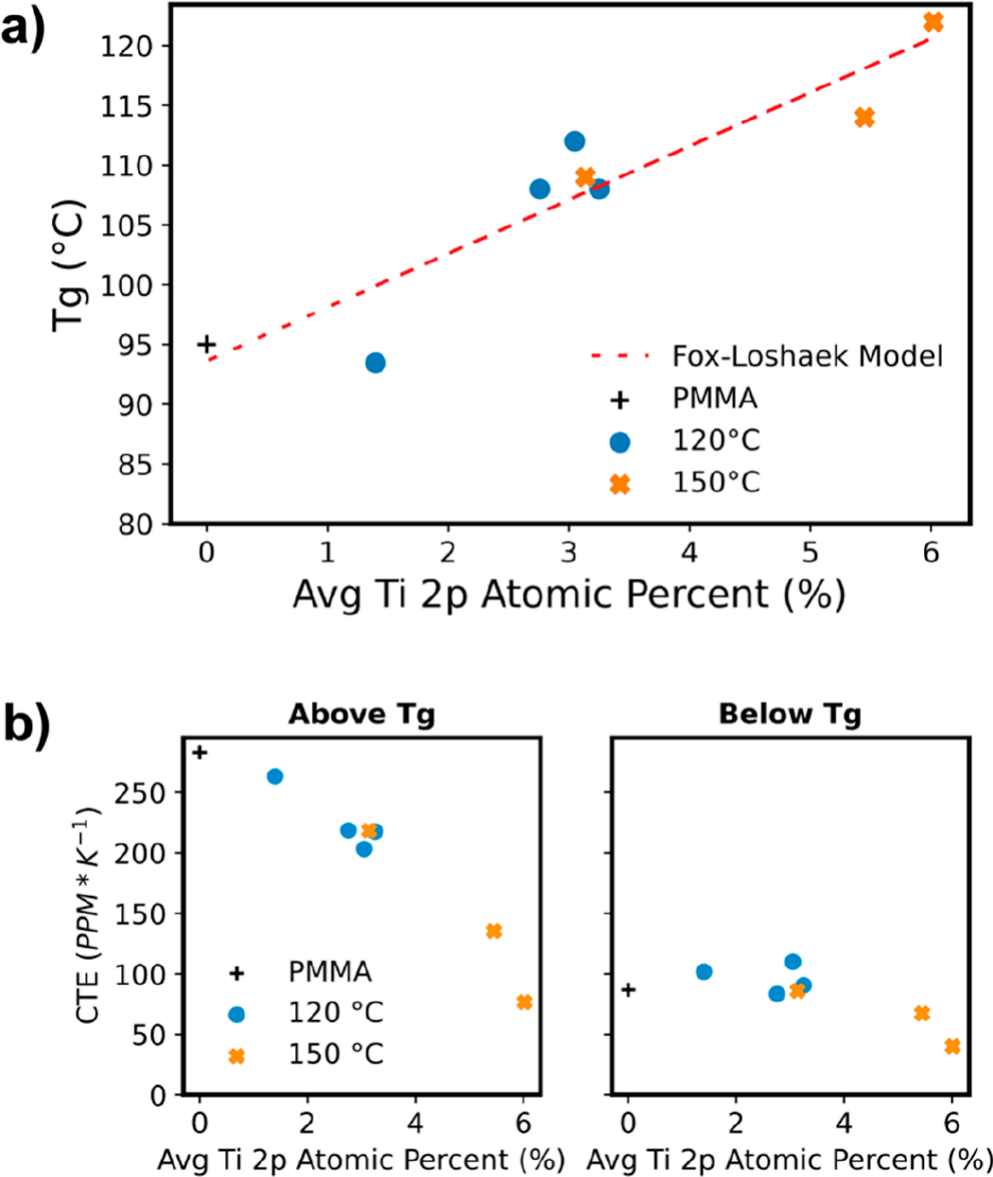
Thermophysical properties of PMMA films infiltrated with TiCl_4_ + H_2_O at 120 and 150 °C: (a) Glass transition temperature and (b) linear coefficient of thermal expansion (CTE) above and below the glass transition temperature. The average TiO_*x*_ concentration is the weighted average of the XPS Ti 2p atomic % throughout the depth of the film. The black “+” shows the values for the untreated PMMA film.

To further test for evidence of chemical cross-linking, the thermophysical properties of these TiO_*x*_–PMMA hybrids are investigated. Using a heated-stage spectroscopic ellipsometer, we quantify both the glass transition temperature (*T*_g_) and coefficient of thermal expansion (CTE) of these materials above and below the *T*_g_.^8,32,33^ Raw data from these measurements are included in Figure S2a,c. Figure 7 summarizes these data by plotting the *T*_g_ and CTE as a function of average inorganic loading for the TiO_*x*_–PMMA hybrids. Figure 7a shows that increasing TiO_*x*_ concentration increases the *T*_g_. Pure PMMA films exhibit a *T*_g_ of about 95 °C, consistent with reports in the literature.^32,48,49^ With increasing TiO_*x*_ loading, the *T*_g_ exceeds 120 °C. This increase in *T*_g_ is consistent with increasing the cross-link density of a polymer.^8,50−52^ As cross-link density increases, the polymer’s chain mobility becomes more restricted, increasing *T*_g._ We can further assess the consistency of *T*_g_ increasing due to increased cross-link density by comparing to the Fox–Loshaek model.^8,50^ Assuming the TiO_*x*_ concentration is correlated to the hybrid’s cross-linking density, the dotted line in Figure 7a shows the best fit to the Fox–Loshaek model. Here, the fitting parameter, *K*_c_, which represents the cross-link’s mechanical stiffness, is found to be 4.2, similar to the value of 3 reported for the inorganic cross-links in the AlO_*x*_–PS-*r*-PHEMA hybrid studied by Bamford et al.^8^ While this value is higher than common organic cross-links, this result may suggest that we are actually forming multiple cross-links from a single TiCl_4_ molecule. TiCl_4_ reacting with multiple ester groups forming bonds to multiple PMMA chains could explain why no chloride ligands remain for the reaction with water, as indicated by the QCM gravimetry data in Figure 4.

Figure 7b plots the linear CTE of these hybrids as a function of the TiO_*x*_ loading. Above *T*_g_ the CTE decreases dramatically with increasing inorganic loading, from a value of 283 ppm/°C for pure PMMA films to 77 ppm/°C for the highest TiO_*x*_ loading fraction. This decrease in CTE is approximately linear with the inorganic loading fraction. This reduction in CTE is again consistent with the formation of chemical cross-links that prevent chain motion and, hence, thermal expansion of the hybrid material. Below *T*_g_ the reduction in CTE is much more modest. In the glassy state, the motion of polymer chains requires a sufficient free volume for atomic motion. Below the glass transition temperature, the density increases, and it becomes increasingly difficult for an atom to find sufficient free volume for motion to occur on a reasonable time scale.^33,53^ Therefore, it is more difficult for the CTE to change substantially below *T*_g_. Additionally, in the glassy state, the thermal energy is already so low that it is difficult to overcome secondary bonds, so it is not until significant quantities of primary cross-links are formed that a noticeable drop in CTE occurs. However, at the highest loadings, CTE still drops by ∼50% compared to the neat polymer.

**Scheme 2:**
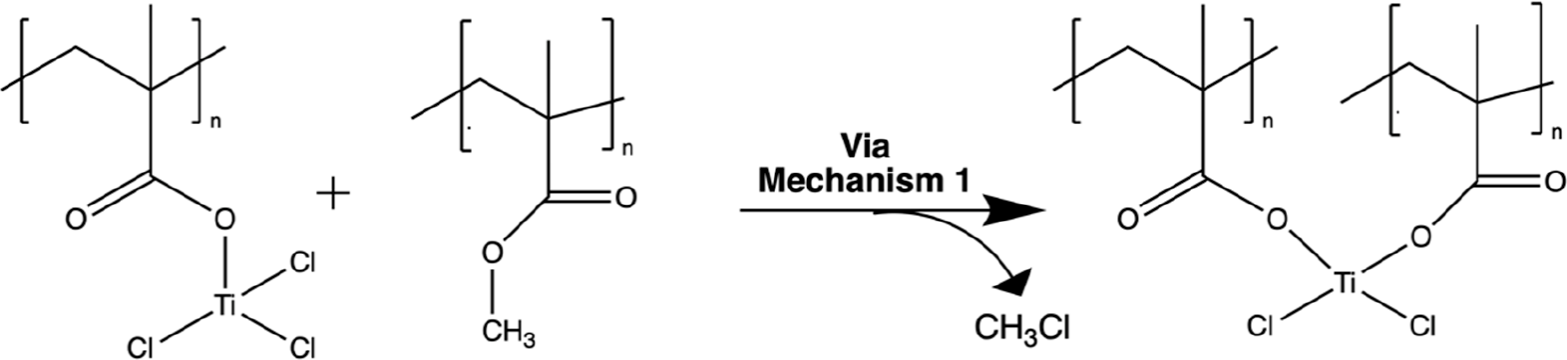
Proposed Mechanism for Inorganic Cross-Linking of PMMA Infiltrated with TiCl_4_ The TiCl_3_ side group product from Scheme 1 continues to act as a Lewis acid that can further propagate concerted dealkylation reactions with nearby PMMA ester groups.

The chemical and thermal properties observed in PMMA–TiO_*x*_ hybrid materials suggest that true chemical cross-links form during infiltration. These observations are consistent with the formation of primary chemical bonds between the organic and inorganic components as evidenced earlier. However, to achieve cross-linking, the final product of Scheme 1 must continue to react. Scheme 2 presents one posited cross-linking pathway where the bound TiCl_3_ species continues to act as a Lewis acid that can undergo a subsequent concerted dealkylation reaction with a neighboring PMMA ester group, presumably on a nearby polymer chain, thus forming a chemical cross-link. While we cannot rule out the possibility of these −TiCl_3_ groups hydrating with water and then forming C–O–Ti–O–Ti–O–C linkages via condensation with nearby metal hydroxides, the apparent lack of reaction with water in the QCM data and the lack of hydroxyls in the FTIR spectra suggest that this mechanism is unlikely. Currently, we suspect that the product in Scheme 2 may continue to react until all chloride ligands react with nearby PMMA chains, such that the single TiCl_4_ molecule actually cross-links with up to four different PMMA chains. This higher-order cross-linking would be consistent with the unusually high stiffness value calculated in the Fox–Loshaek analysis.

## Conclusion

TiCl_4_ infiltration into PMMA appears to occur via a reaction-limited mechanism. This paper elucidates the chemical mechanism of this rate-limiting reaction. Spectroscopically, a C–O–Ti bond forms but not with the PMMA’s carbonyl group, which is undisturbed. Instead, the TiCl_4_ appears to react with the ester group’s methoxy oxygen, via a concerted dealkylation of the methyl side group, removing this methyl group. XPS spectroscopy, especially for O 1s, suggests that the ester O–Me group is consumed during the TiCl_4_ VPI reaction and not the carbonyl. Additionally, QCM gravimetry reveals that after the initial sorptive mass uptake a consistent mass loss occurs during TiCl_4_ infiltration, consistent with the loss of a massive byproduct, presumably chloromethane. Measuring the thermophysical properties of the resultant TiO_*x*_–PMMA hybrids provides further evidence that primary chemical bonds are forming between the organic and inorganic constituents of this material. For example, these hybrids are insoluble in organic liquids that are good solvents for the pure PMMA polymer. Moreover, the glass transition increases with infiltration, and the thermal expansion is reduced. All of these thermophysical observations are consistent with the formation of chemical cross-links. These results thus introduce a new reaction pathway between vapor-phase-infiltrated inorganic precursors and organic polymers. While prior studies have shown direct reactions between precursors and the carbonyl, this is the first time a clear reaction is shown with the methyl–oxy group, leading to cleavage and byproduct generation. The knowledge reported here about the reaction between TiCl_4_ and PMMA will likely inform future investigation for infiltrating a variety of similar polymers with comparable ester functional groups.

## Supporting Information Available

FTIR difference spectra showing PMMA infiltrated with TiCl_4_ at varying temperatures (90, 120, and 150 °C) with 24 h precursor exposure time; plots of raw data collected for hybrid film thickness as a function of temperature used to evaluate the glass transition temperature and the coefficient of thermal expansion of these materials; plot of mass uptake versus time collected via *in situ* QCM gravimetry during VPI of TiCl_4_ + H_2_O into PMMA at 150 °C and subsequent analysis of reaction and byproduct stoichiometries^31^ (PDF)
