# Supplementary material for: Dealkylation of Poly(methyl methacrylate) by TiCl4 Vapor Phase Infiltration (VPI) and the Resulting Chemical and Thermophysical Properties of the Hybrid Material
Source: Chem Mater. 2024 Jan 10;36(2):838–47. doi: 10.1021/acs.chemmater.3c02446 (PMC10809413; doi:10.1021/acs.chemmater.3c02446)
Supplement: Supplementary file 1 — cm3c02446_si_001.pdf [file cm3c02446_si_001.pdf]

Dealkylation of Poly (Methyl Methacrylate) by $TiCl_4$ Vapor Phase Infiltration (VPI) and the resulting chemical and thermophysical properties of the hybrid material

*Shuaib A. Balogun, Sierra S. Yim, Typher Yom, Benjamin C. Jean and Mark. D Losego**
School of Materials Science and Engineering, Georgia Institute of Technology, Atlanta, GA, USA

*Email: losego@gatech.edu
Phone: 404-385-3630

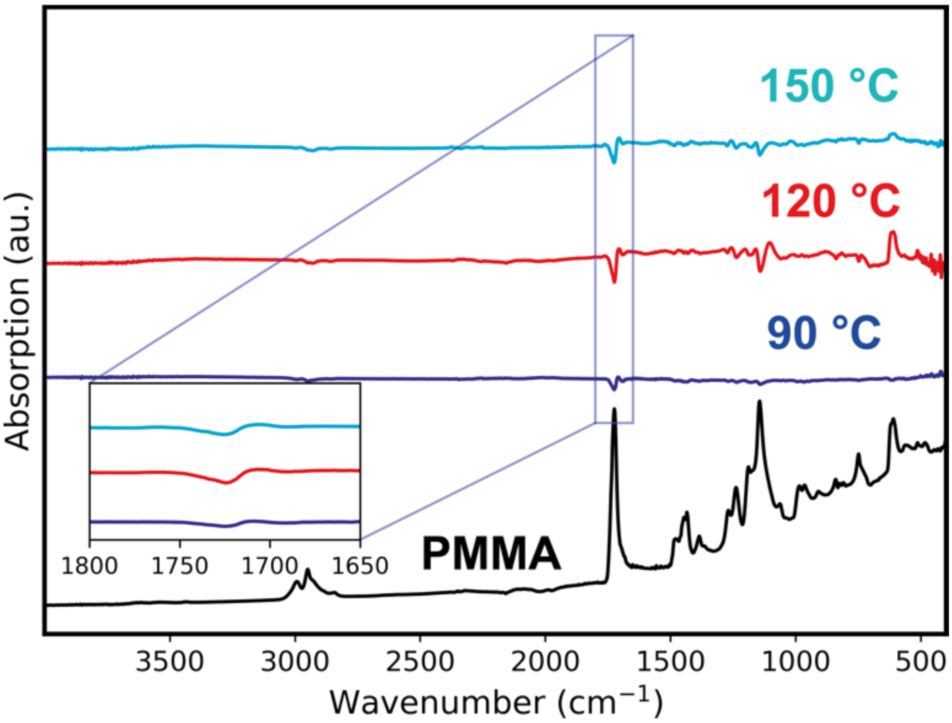

Figure S1: FTIR spectroscopy showing PMMA infiltrated with $TiCl_4$ at varying temperatures (90 , 120 and 150℃) with 24 hour precursor exposure time. Bottom spectrum is for pure PMMA; above are difference spectra computed from the difference between spectra collected for neat PMMA and PMMA infiltrated at varying temperatures. Inset expands the region for the difference spectra of the carbonyl stretch.

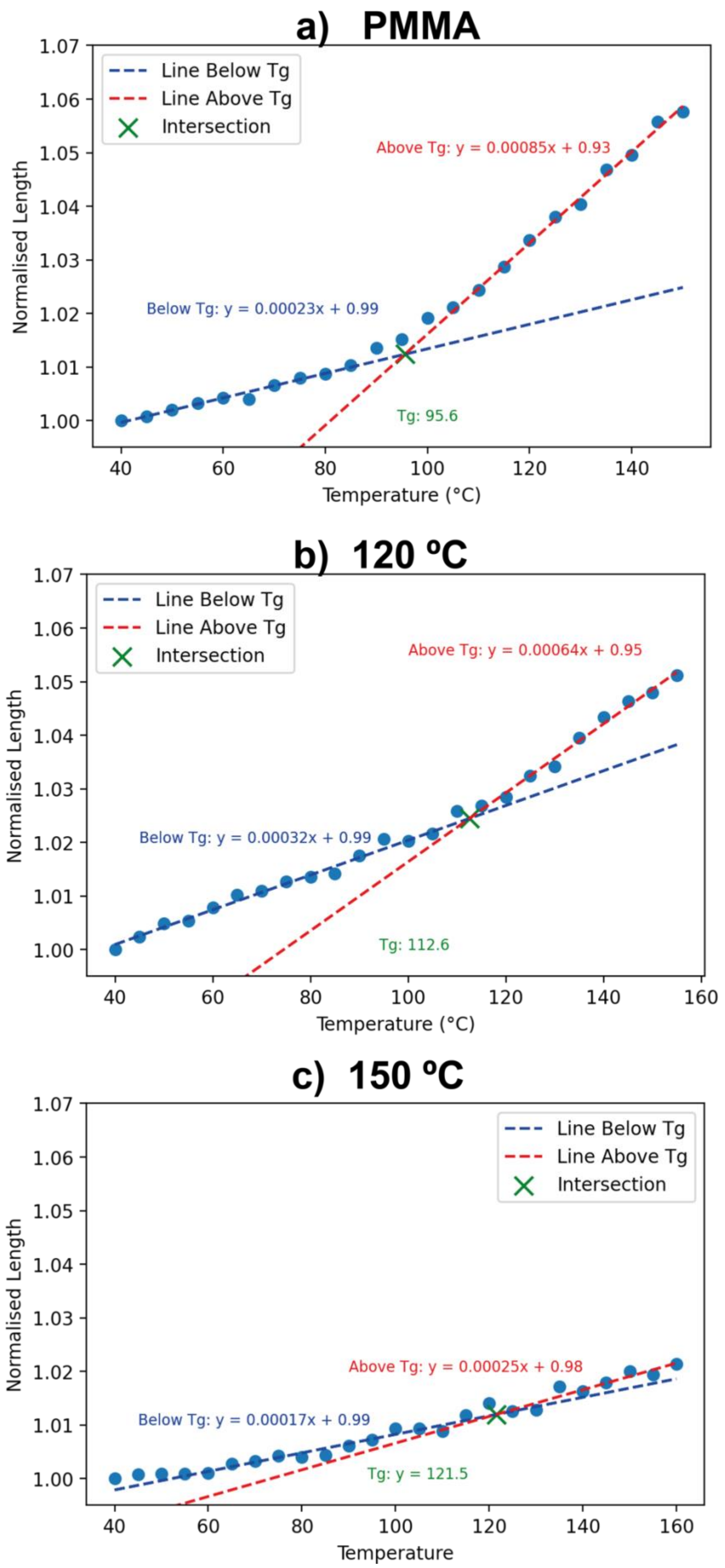
a) PMMA
Line Below Tg
Line Above Tg
Intersection
Above Tg: y = 0.00085x + 0.93
Below Tg: y = 0.00023x + 0.99
Tg: 95.6
Normalised Length
Temperature (°C)
b) 120 ºC
Line Below Tg
Line Above Tg
Intersection
Above Tg: y = 0.00064x + 0.95
Below Tg: y = 0.00032x + 0.99
Tg: 112.6
Normalised Length
Temperature (°C)
c) 150 ºC
Line Below Tg
Line Above Tg
Intersection
Above Tg: y = 0.00025x + 0.98
Below Tg: y = 0.00017x + 0.99
Tg: y = 121.5
Normalised Length
Temperature

Figure S2: Thermal expansion plots showing the change in length with changing temperature for Neat PMMA and PMMA infiltrated at 120 and 150 ℃. The plots show the shift in glass transition temperature at increased processing temperatures. Additionally, the reduction in coefficient of thermal expansion can be observed.

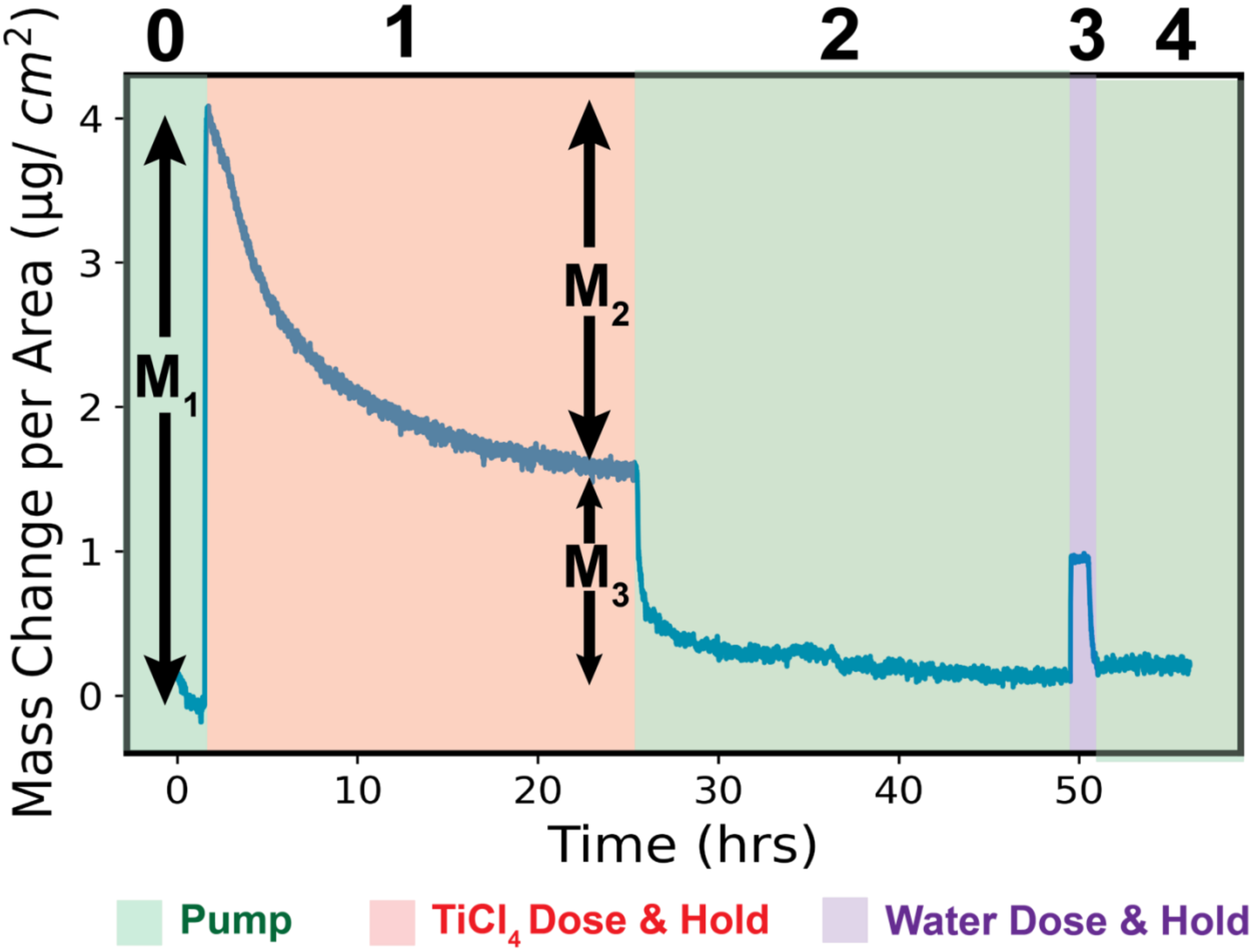

Figure S3: Mass uptake versus time plot collected via *in situ* QCM gravimetry during VPI of $TiCl_4$ + H_2O into PMMA at 150 °C. The plot is separated into five temporal regimes: (0) Pre-infiltration pumping (vacuum base pressure), (1) $TiCl_4$ exposure (3.2 Torr), (2) $TiCl_4$ removal via vacuum pumping, (3) H_2O exposure (12 Torr), and (4) H_2O removal via vacuum pumping. The mass uptake is normalized to the original mass of the polymer (3.24E-05 grams) to provide a percentage of mass added to the polymer via infiltration. All masses are calculated from the Sauerbrey equation.[31] M_1 represent the mass uptake for initial sorption of TiCl4 into the PMMA thin film. M_2 represents the amount of mass lost due to the reaction between $TiCl_4$ and the PMMA functional ester group and M_3 represents ($1.28x10^{-6}$ g) is the unreacted $TiCl_4$ that is simply dissolved in the polymer and desorbs once we remove the TiCl4 overpressure.

Figure S3 shows the mass uptake versus time plot collected via in situ QCM gravimetry during VPI of $TiCl_4$ + H_2O into PMMA at 150 °C. Specifically, it highlights the various mass changes that can be used to determine the reaction and byproduct ratios. M_1 ($4.00x10^{-6}$ g) is the mass uptake for the initial sorption of $TiCl_4$ into the PMMA thin film. M_2 ($2.40x10^{-6}$ g) is presumably the amount of mass lost due to the reaction between $TiCl_4$ and the PMMA functional ester group. Here we note that this mass loss may include a single $TiCl_4$ species

reacting with up to four different ester functional groups. We assume that M_3 ($1.28x10^{-6}$ g) is the unreacted $TiCl_4$ that is simply dissolved in the polymer and desorbs once we remove the TiCl4 overpressure. By subtracting M_3 from M_1, we can get the amount of $TiCl_4$ that reacts with the PMMA and remains entrapped in the hybrid ($M_1 – M_3 = 2.72x10^{-6}$ g). Using its molar mass, this equates to reacting $1.43x10^{-8}$ moles of $TiCl_4$. M_2 is presumably all byproduct release given that the material is still being exposed to a $TiCl_4$ overpressure during this process. Assuming that this byproduct is chloromethane with a molar mass of 50.5 g/mole, this equates to a molar loss of $4.75x10^{-8}$ moles of CH_3Cl. The molar ratio of $TiCl_4$ reacted to CH_3Cl released is 1:3.32. This indicates that each of the entrapped $TiCl_4$ molecules reacts with on average ~3.3 methoxy functional groups or in other words forms ~3.3 bonds to PMMA chains. This value approaches the full reaction extent of 4 bonds, and we suspect steric hindrances limit any further reaction under the process conditions explored.
